# Effect of the Particle Size and Layer Thickness of GNP Fillers on the Dielectric Properties and Actuated Strain of GNP–PDMS Composites

**DOI:** 10.3390/polym14183824

**Published:** 2022-09-13

**Authors:** Jin-Sung Seo, Do-Hyeon Kim, Heon-Seob Jung, Ho-Dong Kim, Jaewon Choi, Minjae Kim, Sung-Hyeon Baeck, Sang-Eun Shim

**Affiliations:** 1 Department of Chemistry and Chemical Engineering, Education and Research Center for Smart Energy and Materials, Inha University, Incheon 22212, Korea; 2 150 Hyundai Research Center, Namyang-eup, Gyeonggi-do, Hwaseong-si 18280, Korea; 3 Department of Polymer Science and Engineering, Kyungpook National University, Daegu 41566, Korea; 4 School of Mechanical and Control Engineering, Handong Global University, 558 Handong-ro, Buk-gu, Pohang Gyeongbuk 37554, Korea

**Keywords:** electro active polymers, dielectric elastomer actuator, polydimethylsiloxane, graphene nanoplatelets, dielectric constant, loss tangent, actuated strain, composite

## Abstract

Dielectric elastomer actuators (DEAs), a type of electroactive polymers (EAPs), are smart materials that are used in various fields such as artificial muscles and biomimetic robots. In this study, graphene nanoplatelets (GNPs), which are conductive carbon fillers, were added to a widely used DEA, namely, polydimethylsiloxane (PDMS), to improve its low actuated strain. Four grades of GNPs were used: H5, H25, M5, and M25 (here, the number following the letter indicates the average particle size of the GNPs in μm). The average layer thickness of the H grade is 13–14 nm and that of the M grade is 5–7 nm. PDMS composites were prepared by adding 0.5, 1, 2, and 3 wt% of each GNP, following which the mechanical properties, dielectric properties, and actuated strain of the composites were measured. The mechanical properties were found to increase as the particle size increased. Regarding the dielectric characteristics, it was found that the higher the aspect ratio of the filler, the easier the formation of a micro-capacitor network in the composite—this led to an increase in the dielectric constant. In addition, the higher amounts of GNPs in the composites also led to an increase in the dielectric constant. For the actuated strain analysis, the electromechanical sensitivity was calculated using the ratio of the dielectric constant to the Young’s modulus, which is proportional to the strain. However, it was found that when the loss tangent was high, the performance of the actuated strain decreased owing to the conversion of electric energy into thermal energy and leakage current loss. As a result, the highest actuated strain was exhibited by the M25 composite, with an actuated strain value of 3.01% measured at a low electric field (<4 kV/mm). In conclusion, we proved that the GNP–PDMS composites with a thin layer and large particle size exhibited high deformation.

## 1. Introduction

**Figure 1:**
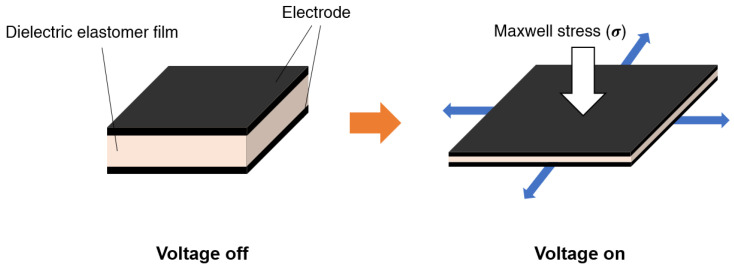
Working principle of the dielectric elastomer actuator.

Extensive attempts are currently being made to solve a variety of engineering problems in the automobile, machinery, robot, aviation, and medical industries by using smart materials whose characteristics can be altered by external stimulation [1,2,3]. Among them, electroactive polymers (EAPs) possess many advantages, such as large deformation, low power consumption, low cost, good processability, and fast response [4,5,6,7,8]. EAP can contract, expand, and bend with electrical stimulation; they can therefore be used in the field of actuators and generators because of their ability to convert electrical and mechanical energy into each other [8,9,10]. Unlike other materials, EAPs have a similar strength to muscles, allowing for their application in fields such as artificial muscles [11,12,13], biomimetic robots [14], medical devices, and tactile displays [15,16]. Representative materials include polyvinylidene fluoride (PVDF), polyvinyl alcohol (PVA), poly(pyrrole) (PPy), polyaniline (PANI), poly(ionic liquids), polymer–metal composites, etc. Dielectric elastomers are a type of EAP which offer various advantages in terms of electromechanical characteristics, flexibility, dielectric constant, and reactivity; thus, they are the most widely used of the EAPs [17,18,19,20,21]. However, the main limitation of dielectric elastomers is their requirement for a high driving voltage (>10 kV/μm) for high deformation, which is unlike other EAPs, such as conductive polymers [22,23], ionic polymer gels [24], ferroelectric polymers [25,26,27,28] and liquid-crystal elastomers [28,29,30]. Many researchers have attempted to lower the driving voltage of DE; however, industrial application remains challenging. Dielectric elastomeric actuators (DEAs) consist of dielectric elastomer films coated with flexible conductive electrodes on both sides. Carbon materials with high conductivities, such as carbon nanotubes (CNTs), carbon grease, and graphite grease, are commonly used as the flexible electrodes [31,32,33,34]. When a voltage is applied between both electrodes, polarization occurs in the polymer matrix, generating a Maxwell stress (σ). The DEA is compressed in the thickness direction and expands in the plane direction due to the Maxwell stress (σ) [35,36,37]. When the voltage is removed, it returns to its original form (Figure 1).

The Maxwell stress formula when voltage is applied is shown in Equation (1):

(1) $$\sigma \;\;=\varepsilon_{r}\varepsilon_{0}E^{2}$$

where $\varepsilon_{r}$ is the dielectric constant of the elastomer, $\varepsilon_{0}$ is the dielectric constant of free space ($8.85\times 10^{-12}{\;\mathrm{Fm}}^{-1})$, and *E* is the applied electric field. The planar strain, a value representing the DEA deformation, can be obtained using the displacement caused by the Maxwell stress in the thickness direction.

The formula for determining the plane direction displacement is given in Equation (2):

(2) $$S_{z}\;=-\frac{\sigma }{Y}=-\frac{\varepsilon_{r}\varepsilon_{0}E^{2}}{Y}=-\varepsilon_{0}\beta E^{2}$$

The formula for determining the planar strain is shown in Equation (3):

(3) $$S_{p}=\frac{1}{1+S_{z}}-1\;$$

where Y is the Young’s modulus of the material, and β is the electromechanical sensitivity ($\beta =\varepsilon_{r}/Y$). Electromechanical sensitivity ($\beta =\varepsilon_{r}/Y$) is the most important property of DEA and can have a large deformation value when the dielectric constant is high and Young’s modulus is low. For a large deformation of the dielectric elastomer, the dielectric constant of the material must be high. The general method of achieving this involves increasing the interfacial polarization by adding micro-capacitors to the matrix.

The matrix is one of the most important factors in the DEA. The matrix must have satisfactory values for various characteristics, such as stability, stiffness, Young’s modulus, and dielectric constant, to improve the actuated strain. Polydimethylsiloxane (PDMS) is one of the most commonly used matrices because of its high reactivity, low Young’s modulus (<1 MPa), low stiffness, low price, and excellent moldability. However, PDMS, being a non-polar rubber, has the critical disadvantage of having a low dielectric constant, which acts as a limitation when used in DEAs. Therefore, it is essential to increase the dielectric constants of DEA materials. The dielectric constant can be increased by adding fillers, such as ceramic fillers (BaTiO_3_, CCTO, TiO_2,_, Al_2_O_3_, and HfO_2_) [38,39,40,41,42,43] and conductive carbon fillers (graphene, CNT, carbon black, and graphite) [44,45]. Ceramic fillers were frequently used in the past; however, they can provide a high dielectric constant only when a large amount of filler is used, which decreases the flexibility of the composite. Among them, graphene is a promising candidate because of its high dielectric constants and good thermal and mechanical properties when added in small amounts. However, when the amount of filler nears a threshold value (the percolation threshold), transition from insulator to conductor occurs, and the loss tangent value tends to soar. In contrast, graphene nanoplatelets (GNPs) are widely commercialized because they are less expensive than graphene, and their size and layer thickness can be easily controlled.

Although there are a few papers confirming the increase in permittivity by adding GNP to LDPE [46] and NR [47], there is no study reported to evaluate the effect of loading GNP onto a PDMS matrix on the increase in dielectric properties or actuated strain. In this work, we employed four types of GNPs with different sizes and layer thicknesses added to PDMS, and the changes in mechanical and dielectric properties and actuated strain are comprehensively studied. Actuated strain, in particular, achieved a maximum of 3% at 3 kV/mm by adding as low as 3 wt% of GNP. This actuated strain has potential use in spring rolls, grippers, and insect-like walking robots, among others [48]. We hope this work can serve as a reference point to enlighten researchers to improve electromechanical properties of composites by adapting the morphological feature of materials.

## 2. Materials and Methods

### 2.1. Materials

Polydimethylsiloxane (SILASTIC^TM^ RBL-9101) with a density of 1.05 g/cm^3^ and standard curing temperature of 120 °C was purchased from Dow Corning (Seoul, Korea). GNPs of grades M5, M25, H5, and H25––the number following the letter denotes the average particle size of the GNPs in μm––were provided by XG Sciences (Lansing, MI, USA). The particle sizes for each grade were 5 μm and 25 μm. The average layer thickness was 6–8 nm for grade M and 15 nm for grade H; the surface area was 120–150 m^2^/g for grade M and 80 m^2^/g for grade H. Carbon conductive grease with a thermal conductivity of 0.29 W/(m·K), resistance of <400 Ω, and viscosity of 630,000 Cp was purchased from MG Chemicals (Seoul, Korea).

### 2.2. Method

#### 2.2.1. Fabrication of GNP–PDMS Composite

**Figure 2:**
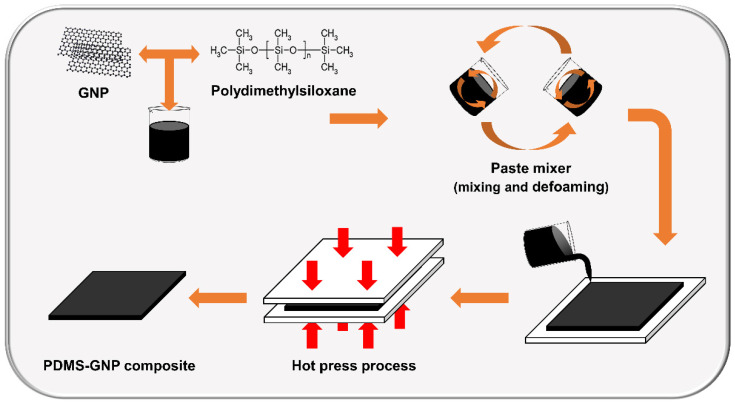
A compounding process to fabricate PDMS–GNP composites.

The method of synthesis of the PDMS–GNP composites is illustrated in Figure 2. The sample was prepared using a paste mixer (PDM 300, Daewha Tech, Yongin, Korea) in which, through revolution and rotation, the GNPs were uniformly dispersed in the PDMS; air bubbles were removed from the mixture through a defoaming process. The PDMS (SILASTIC^TM^ RBL-9101) used here consists of part A and part B. PDMS elastomer was prepared by mixing A and B in a ratio of 1:1. GNPs-filled PDMS was fabricated by mixing 55 g of PDMS with GNP (0.5, 1, 2, and 3 wt% of PDMS) to make elastomer films. When using the paste mixer, the uniform mixing process was used for 20 min at 1500 rpm for revolution and 0 rpm for rotation. After that, the defoaming process was performed at 1500 rpm for revolution and 150 rpm for rotation for 15 min. Then, the mixture was poured into a dielectric sample mold (5 × 5 mm^2^ area and 2 mm thickness), a mechanical sample mold (15 × 15 mm^2^ area and 2 mm thickness), and an actuating sample mold (15 × 15 cm^2^ area and 250 μm thickness). The curing process was conducted using a hot press (DHMP-10S, Dongjin Machinery, Incheon, Korea). The curing condition of the dielectric and mechanical sample molds was 130 °C for 3000 s, and the curing condition of the actuating sample mold was 130 °C for 1000 s.

#### 2.2.2. Characterization

The crystal structure, phase, and crystal orientation of the GNPs were examined via X-ray diffraction (X’pert Pro MRD, Malvern PANalytical, Malvern, UK).

The particle size and layer thickness of the GNPs were measured by field-emission transmission electron microscopy (FE-TEM; JEM-2100F, JEOL, Tokyo, Japan). When the sample and grid are of the same carbon series, the images may overlap; therefore, a holey gold grid (CF313-50, Electron Microscopy Sciences, Hatfield, PA, USA) was used.

The particle size and layer thickness of the GNPs were obtained using an atomic force microscope (Nanoscope Multimode IV a, Bruker, Billerica, MA, USA); a sample was prepared by spin coating after GNPs that were diluted in water were placed on a silicon wafer.

Tensile strength (ASTM D412) and elongation at break were measured using a universal testing machine (DUT-2TC, Daekyoung Engineering Co., Bucheon, Korea). The load cell used was 20 kgf, and the crosshead speed was 500 mm/min, averaged after five measurements for achieving sufficient accuracy. 

The dielectric constant and loss tangent were measured using an LCR meter (4284A, HP/Agilent, Santa Clara, CA, USA) and dielectric test fixture (16415 B, HP/Agilent, Santa Clara, CA, USA). The test frequency was 20–100,000 Hz at room temperature, the point was 100, and the number of repeats per step was 5. The dielectric constant of a sample can be determined as follows:

(4) $$\varepsilon_{r}=\frac{t_{a}\times C_{p}}{A\times \varepsilon_{0}}=\frac{t_{a}\times C_{p}}{\pi \times {\left(\frac{d}{2}\right)}^{2}\times \varepsilon_{0}},\;\;\;\;\;\;\;\;\;\;\;\;\;\;\;\;\;\;\;\;\;D_{t}=D$$

where $C_{p}$ is the equivalent parallel capacitance (F), $t_{a}$ is the average thickness of the test material, A is the electrode area, and $D_{t}$ is the loss tangent of the test materials.

Actuated strain measurements were performed using a continuous function generator (33220A, Agilent, Santa Clara, CA, USA). Experimental conditions were an alternating current (AC) with a minimum applied voltage of 400 V, a maximum applied voltage of 2.6 kV, and a frequency of 1 Hz. As shown in Figure S1, the experimental type was selected as a circular actuated strain test; the movement was confirmed through a laser sensor. An external voltage was applied using an electrode (carbon conductive grease) and copper tape (Figure S1).

## 3. Results and Discussion

### 3.1. Morphology and Characterization of GNPs

**Figure 3:**
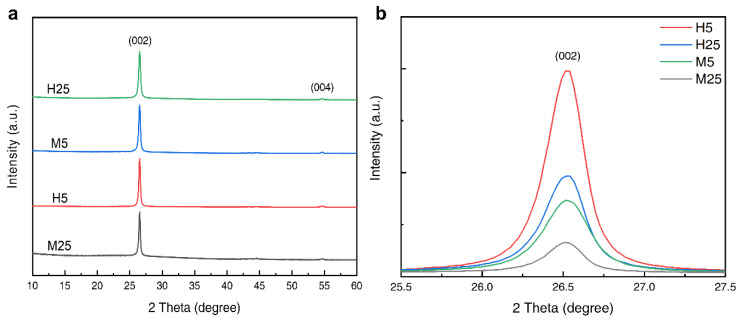
(**a**) The XRD patterns for each grade (H5, H25, M5, and M25) of GNPs, and (**b**) the enlarged portion of the peak (2θ = 26.6°).

The XRD spectra of the four grades of GNPs (H5, H25, M5, and M25) are shown in Figure 3. As seen in Figure 3a, two peaks were observed for each grade of GNP. The first peak (2θ = 26.6°) is sharp and strong, while the second peak (2θ = 54.6°) is relatively weak. The diffraction planes (Miller indices) (002) and (004) are the basal planes of graphene, of which the 2θ = 26.6° peak (002) corresponds to graphite. Figure 3b shows that the width (full width at half maximum) at the peak corresponding to 2θ = 26.6° is different for each grade of GNP. It is known that the wider the peak, the smaller the particle size. The graph shows that the peak width of H5 was wider than that of H25, and the peak width of M5 was wider than that of M25. These results confirm that the particle sizes of H5 and M5 were smaller than those of H25 and M25. In addition, it can be observed that the height of the peak is different for each grade; this is related to their layer thickness. The layer thicknesses of the GNPs specified by the manufacturer are 14 nm and 7 nm for grades H and M, respectively. The peaks of the grade M GNPs, which have a thinner layer thickness, are lower than the peaks of the grade H GNPs; therefore, it can be inferred that the thinner the layer thickness, the lower the height of the peak.

**Figure 4:**
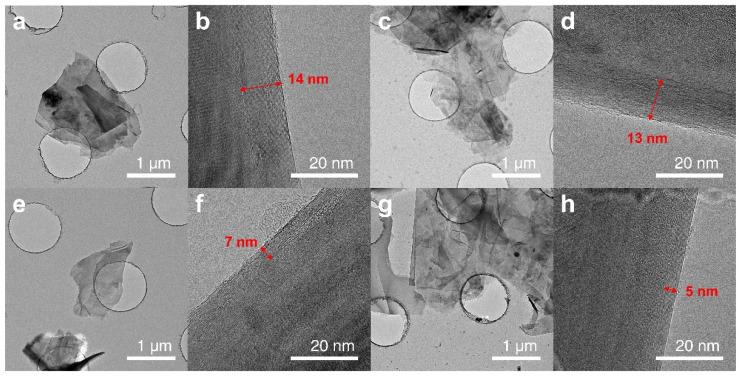
TEM microphotographs of (**a**,**b**) H5, (**c**,**d**) H5, (**e**,**f**) M5, and (**g**,**h**) M25. Left images (**a**,**c**,**e**,**g**) show the particle size and right images (**b**,**d**,**f**,**h**) show the layer thickness of GNPs.

The TEM micrographs based on the grade of the GNPs are shown in Figure 4. Figure 4a,c,e,g show the top parts of the GNP, and Figure 4b,d,f,h show the side parts. In the TEM images of the top parts of the GNPs, several GNPs of different sizes are found to be overlapped; folds can be observed at the edge, and small pieces of GNPs broke during the fabrication process. Figure 4a,e show GNPs with particle sizes of 5 μm and Figure 4c,g of 25 μm, respectively. In the TEM images of the top view, the particle sizes in Figure 4c,g are larger than those in Figure 4a,e. Figure 4b,d,f,h show side-view TEM images confirming the folded part of the edge of GNPs. Furthermore, the graphene sheet is evidently stacked. It was confirmed that Figure 4b,d are grade H, with a layer thickness of 13–14 nm owing to the stacking of approximately 40–50 layers, and Figure 4f,h are grade M, with a layer thickness of 5–7 nm owing to the stacking of approximately 17–23 layers.

### 3.2. Mechanical Properties of GNP–PDMS Composites

**Figure 5:**
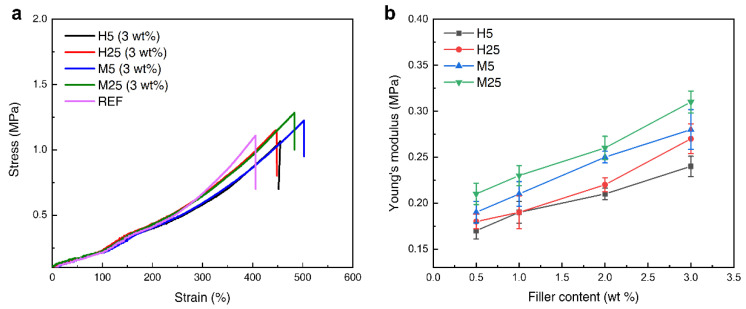
Stress–strain curve (**a**) for each grade at filler contents of 3 wt%, Young’s modulus (**b**) for each grade according to filler contents.

The stress–strain curves and Young’s moduli of the GNP–PDMS composites are shown in Figure 5. Figure 5a shows the stress–strain behaviors of the PDMS composites containing the four grades of GNPs at the same content of 3 wt%. The tensile strength of the M25 PDMS was 1.3 MPa, which indicates an increase of approximately 18% compared with the tensile strength of neat PDMS (i.e., 1.1 MPa). If a filler is uniformly well-dispersed in the PDMS matrix, the stress transfer from the PDMS to the filler improves, thus enhancing the mechanical properties of the composite. The tensile strengths of the PDMS composites containing M5 and H25 increased by 9% and 4.25% to 1.2 and 1.15 MPa, respectively; however, the tensile strength of the composite containing H5 decreased by 3.6% to 1.06 MPa. This shows that the mechanical properties of M-grade GNPs consisting of a thinner GNP layer are better than those of the H-grade GNPs. Compared with GNPs with a particle size of 25 μm, the PDMS composites containing GNPs with a 5 μm particle size resulted in a marginal increase or decrease in mechanical properties. This is because the GNPs with a particle size of 5 μm cause substantial agglomeration inside the PDMS composite. This agglomeration gradually increases, causing damage at the GNP–PDMS interface and inducing stress concentration at the agglomerate. This facilitates crack initiation and propagation, which reduces the stress transfer efficiency, thereby reducing the tensile strength. Figure 5b shows the Young’s modulus graph for each grade content. As the content of GNP increased, Young’s modulus increased proportionally. The largest increase, as compared with the neat PDMS composite, occurred for the M25 grade GNPs, whose Young’s modulus (0.31 MPa) was higher by 72.2%.

### 3.3. Electromechanical Properties of GNP–PDMS Composites

**Figure 6:**
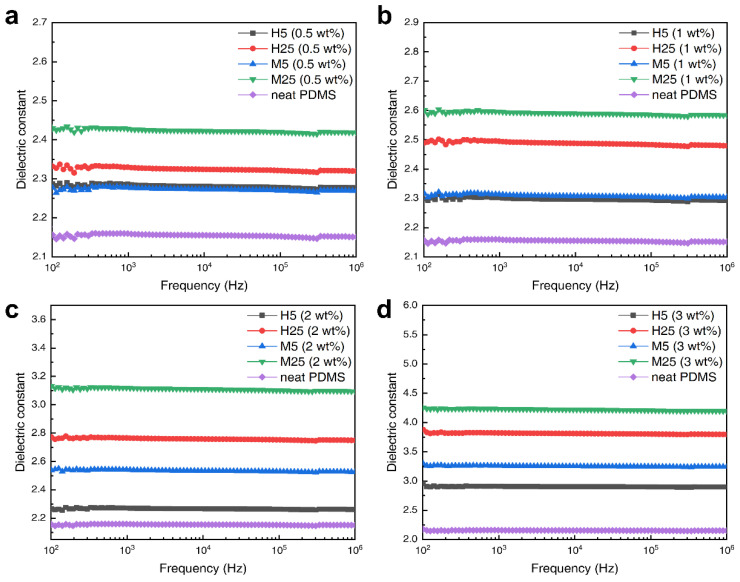
Dielectric constant of GNP composites graph according to filler contents; (**a**) 0.5, (**b**) 1, (**c**) 2, and (**d**) 3 wt% graphene.

Figure 6 shows the dielectric constants of the GNP–PDMS composites according to the frequency change. The graphs demonstrate that as the amount of GNP increased, the dielectric constants of the PDMS composites also increased proportionally. As seen in Figure 6a, when the GNP content is 0.5 wt%, the dielectric constants of the composites are only slightly higher than that of the neat PDMS. However, as shown in Figure 6d, a significant increase in the dielectric constants was observed when the GNP content increased to 3 wt%. Compared with the dielectric constant of neat PDMS (2.15), the dielectric constant of the M25–PDMS composite was 4.26, exhibiting a nearly two-fold increase. The main reason for this change in the dielectric constant with the addition of GNP to PDMS is the formation of a micro-capacitor network structure. GNPs can form micro-capacitors more easily due to their plate-shaped structural characteristics. When plate-shaped GNPs are positioned at both ends in parallel or in series with the PDMS matrix interposed, a micro-capacitor structure is formed, which accumulates multiple charge carriers at the matrix internal interface, increasing the interfacial polarization sites and improving dielectric properties. In addition, if multiple micro-capacitors are created to form a network structure connected to each other, a higher dielectric constant increase can be achieved. For this reason, adding more GNPs to PDMS increases the micro-capacitor network inside the PDMS matrix, and the dielectric constant increases proportionally to the GNP content. In addition, the M-grade GNP–PDMS composite was found to be more effective at increasing the dielectric constant. The main reason why the different layer thicknesses of the fillers changed the dielectric constants of the composites can be explained using the aspect ratio of the GNPs. For the same GNP content, the filler with a higher aspect ratio is more advantageous for creating a micro-capacitor network in the composite than a filler with a lower aspect ratio. The aspect ratios calculated by the ratio of the particle size to layer thickness were 370.3, 1851.8, 833.3, and 4166.6 for H5, H25, M5, and M25, respectively. The M25 grade GNPs, which had the thinnest layer and largest diameter, showed the highest aspect ratio of 4166.6. In contrast, the H5 grade GNPs with the thickest layer and smallest diameter had the lowest aspect ratio at 370.3. As indicated by Table 1, the dielectric constant of the H5 composite was 2.9, while those of the other composites were 3.42, 3.82, and 4.26 for the M5, H25, and M25 composites, respectively. This result confirms that the higher aspect ratio of the filler, the higher would be the dielectric constant.

**Figure 7:**
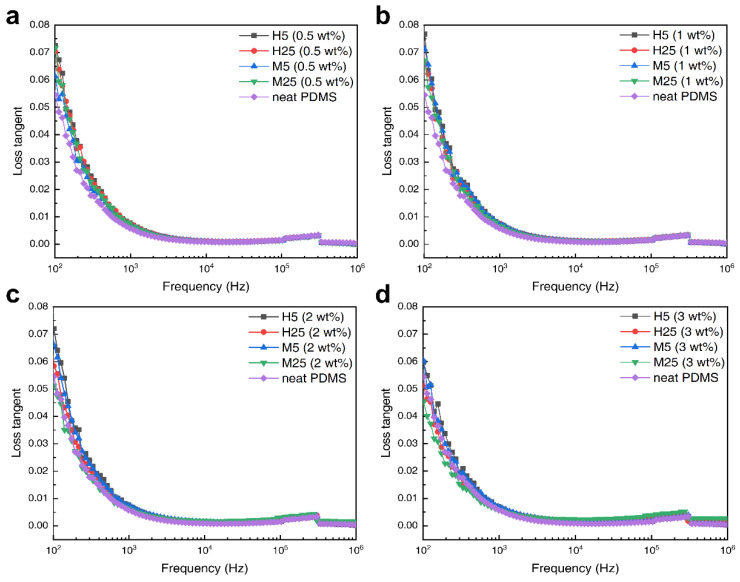
Loss tangent of GNP composites graph according to filler contents; (**a**) 0.5, (**b**) 1, (**c**) 2, and (**d**) 3 wt% graphene.

Figure 7 illustrates the dependence of the loss tangent on the frequency of the composites with different amounts (0.5, 1, 2, and 3 wt%) of the four grades of GNP. The dielectric loss tangent can be calculated using the ratio of the imaginary part (ε″) to the real part (ε′). In the low-frequency region (1–10^3^ Hz), all the composites show a sharp decrease in the loss tangent as the frequency increases; this is because the interfacial polarization between PDMS and GNP cannot keep up with the rate of change in frequency. In contrast, in the high-frequency region (10^4^–10^6^ Hz), the loss tangent increases as the frequency increases because of dipole relaxation.

As aforementioned, the GNPs create a micro-capacitor network structure inside the composite, increasing the dielectric constant. When a certain number of GNPs is reached, overlapped micro-capacitor network structures connect to each other and form a conductive pathway. When an external field is applied, the leakage current flows through this conductive pathway, resulting in a surge in the loss tangent and the dielectric constant. The number of GNPs for which this phenomenon occurs is called the percolation threshold. In Figure 6 and Figure 7, there is no region in which the dielectric constant and loss tangent suddenly increase. This supports the fact that the GNP content inside the PDMS had not yet reached the percolation threshold. To achieve a high dielectric constant, the amount of filler added should be as close to the percolation threshold as possible; however, because the loss tangent also increases rapidly at the same time, it is important to adjust the dielectric properties by adding an appropriate amount of filler. In the case of dielectric elastomers, the loss tangent is a crucial factor; therefore, it is recommended to determine an optimum point under the percolation threshold at which an increase in the dielectric constant can be achieved while minimizing the loss tangent.

**Figure 8:**
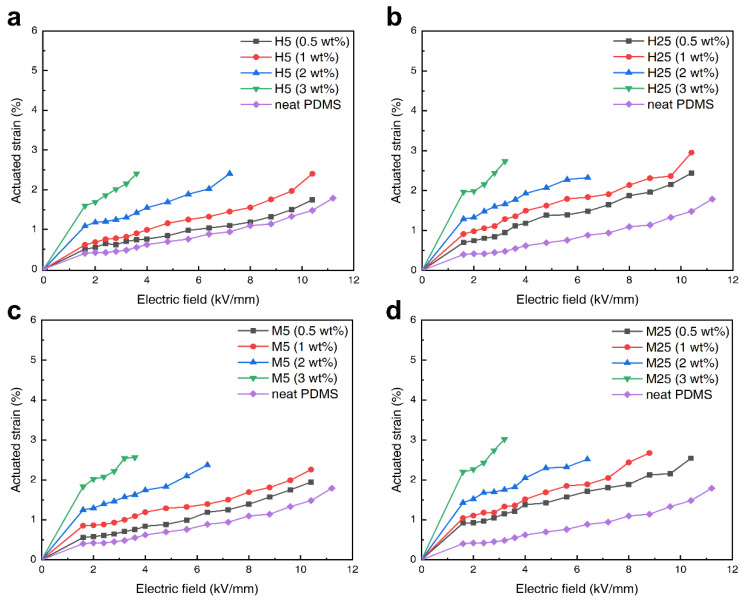
Actuated strain of GNP composites according to filler contents (circular test); (**a**) 0.5, (**b**) 1, (**c**) 2, and (**d**) 3 wt% graphene.

Figure 8 shows the actuated strain of the PDMS composite in which the four contents of the four grades of GNPs were added. As shown in Figure S2, the thickness strain $\left(S_{z}\right)$ measured by the laser sensor (a laser doppler vibrometer) is substituted in Equations (2) and (3) to calculate the plane strain $\left(S_{p}\right)$. The actuated strain test was conducted using a circular test, which is the most representative measurement method. The applied voltage was AC at a frequency of 1 Hz. As shown in Figure 8a–d, PDMS composites with 2 wt% and 3 wt% GNP were destroyed in a high electric field (>4 kV/mm), whereas the neat PDMS as well as 0.5 and 1 wt% GNP composites could be actuated up to a high electric field. This is because defects are more easily formed when high amounts of filler are added to the PDMS, which might cause electrical shorts in the samples. PDMS composites with lower GNP contents can withstand higher applied voltages but have the disadvantage of low actuated strains. However, adding high amounts of filler results in high actuated strains even at low applied voltages. The predominant reason as to why DEA is difficult to apply industrially is its high driving voltage; this problem can be solved with the addition of conductive GNPs, which lower the driving voltage. The electromechanical sensitivity of the materials (listed in Table 1) is one of the most important factors in determining the actuated strain. It is proportional to the ratio of the dielectric constant to the Young’s modulus; therefore, the dielectric constant must be increased, and the Young’s modulus must be decreased to achieve a high actuated strain. Among the four GNP–PDMS composites, the H25 composite exhibited the highest electromechanical sensitivity of 14.14. The dielectric constant was lower than that of the M25 composite; however, it had a lower Young’s modulus, resulting in a higher electromechanical sensitivity. However, as can be seen in Figure 8b,d, when comparing the actuated strains of the PDMS composites with 3 wt% GNPs, the M25 composite showed a higher strain (3.01%) than the H25 composite (2.73%). The reason for this result, despite the H25 composite having higher electromechanical sensitivity, stems from the H25 composite having a higher loss tangent than the M25 composite. If the loss tangent is high, the electric energy is converted to leakage current loss and thermal energy, leading to a lower actuated strain. As a result, the composite with the highest actuated strain at a low applied voltage (<4 kV/mm) was the M25–PDMS composite, for which the maximum strain was measured to be 3.01%, approximately 2.6 times that of the neat PDMS (1.15%).

## 4. Conclusions

In this study, the composites examined were manufactured by adding the conductive carbon filler GNP to PDMS to achieve increased dielectric constant and actuated strain for the composite. Four grades of GNP (H5, H25, M5, and M25) were used to investigate the effects of their different particle sizes and layer thicknesses on the mechanical and dielectric properties of the composites. It was confirmed that the addition of GNPs improved the mechanical and dielectric properties of the composites, ultimately leading to high actuated strains at low voltages. In the mechanical characteristic analysis, the highest enhancement of tensile strength was observed in the M25 composite, whose 1.3 MPa tensile strength indicated an 18% increase compared with the tensile strength of neat PDMS. When 3 wt% of fillers were added, the Young’s modulus of the M25 composite was measured to be approximately 0.33 MPa, which was 83.3% higher than that of the neat PDMS. Consequently, we demonstrated that composites of M-grade GNP, which have a thinner layer, have higher tensile strengths and Young’s moduli than the composites of H-grade GNP. It was observed that the larger the aspect ratio of the filler, the more micro-capacitor network structures that could be formed inside the PDMS composite, resulting in a higher dielectric constant. Foreach GNP, i.e., H5, H25, M5, and M25, the aspect ratios calculated were 370.3, 1851.8, 833.3, and 4166.6, respectively, and the dielectric constants of the 3 wt% composites were 2.9, 3.82, 3.42, and 4.26, respectively. The most effective GNPs for increasing the dielectric constant was determined to be the M25 grade. The most important analysis for the DEA, the actuated strain analysis, was conducted using the circular test, which is the most commonly used test. The electromechanical sensitivity values of the four grades of GNP composites (H5, H25, M5, and M25) were 12.08, 14.14, 12.21, and 13.74, respectively, with the highest value belonging to the H25 composite. However, because it had a higher dielectric loss than the M25 composite, the maximum strain occurred in the M25 composite (3.01%).

In conclusion, this study demonstrated that the addition of GNPs with relatively thin layers and large particle sizes afford high dielectric constants, high mechanical strengths, and actuated strain performances.

## Figures and Tables

**Table 1:** Mechanical Properties and Dielectric Properties of PDMS Composite with 3 wt% GNP.

Sample Label	Tensile Strength (σ)	Elongation at Break (%)	Young’s Modulus (Y)	Dielectric Constant at 1 kHz $\left(\varepsilon_{r}\right)$	Loss Tangent at 1 kHz (δ)	Electromechanical Sensitivity $\left(\beta =\varepsilon_{r}/Y\right)$
Neat PDMS	1.108	406.1	0.18	2.15	0.0053	11.9
H5 (3 wt%)	1.067	451.8	0.24	2.9	0.0062	12.08
H25 (3 wt%)	1.151	448.65	0.27	3.82	0.0061	14.14
M5 (3 wt%)	1.225	502.8	0.28	3.42	0.0059	12.21
M25 (3 wt%)	1.301	483.65	0.31	4.26	0.0054	13.74

## Data Availability

Not applicable.

## Supplementary Materials

The following supporting information can be downloaded at https://www.mdpi.com/article/10.3390/polym14183824/s1, Figure S1: DEA circular test of PDMS/GNP sample using a laser sensor (laser doppler vibrometer), Figure S2: Deformation at maximum voltage of PDMS with 3 wt% addition of 4 grade GNPs measured with a laser doppler vibrometer.

## References

[1] Shahrzad J.M., Alireza M., Carlo M., Rodney G.V. A review of mechanically reconfigurable antennas using smart material actuators. Proceedings of the 5th European Conference on Antennas and Propagation (EUCAP).

[2] Chad B., Justin D., Andrew G., Mark K. (2007). Review of Smart Material Based Actuators for Artificial Muscles. Ph.D. Thesis.

[3] Sun j., Guan Q., Liu Y., Leng J. (2016). Morphing aircraft based on smart materials and structures: A state-of-the-art review. J. Intel. Mater. Syst. Struct..

[4] Ning C., Zhou Z., Tan G., Zhu Y., Mao C. (2018). Electroactive polymers for tissue regeneration: Developments and perspectives. Prog. Polym. Sci..

[5] Damilola R., Tania B., Jennifer A.I. (2019). Biomedical application of electroactive polymers in electrochemical sensors: A review. Materials.

[6] Cohen Y.B., Anderson I.A. (2019). Electroactive polymer (EAP) actuators—Background review. Soft Mater..

[7] Alsksey V.M., Tarek D., Dmitry V.T., Alexander Y.G. (2022). Electroactive polymer-based composites for artificial muscle-like actuators: A review. J. Nanomater..

[8] Asaka K., Mukai K., Sugino T., Kiyohara K. (2013). Ionic electroactive polymer actuators based on nano-carbon electrodes. Science.

[9] Palakodeti R., Kessler M.R. (2006). Influence of frequency and prestrain on the mechanical efficiency of dielectric electroactive polymer actuators. Mater. Lett..

[10] Alici G., Geoffrey M., Spinks J.D., Madden J.D., Yanzhe W., Gordon G. (2008). Response characterization of electroactive polymers as mechanical sensors. IEEE ASME Trans. Mechatron..

[11] Adelyne F., Rauno T., Giao T.M.N., Laurent C., Elisabeth L., John D.W.M., Frederic V., Cedric P. (2018). Linear artificial muscle based on ionic electroactive polymer: A rational design for open-air and vacuum actuation. Adv. Mater. Technol..

[12] Xu H., Han C., Liu X., Li J., Sun Z. (2021). A highly flexible and stretchable ionic artificial muscle. Sens. Actuators A Phys..

[13] Liu X., Xu H., Li Y., Jing M., Wang W., Li Z., Zhang P., Sun Z. (2022). A stretchable and self-healing ionic artificial muscle modified by conductive substances. Appl. Phys..

[14] Chang L., Liu Y., Yang Q., Yu L., Liu J., Zhu Z., Lu P., Wu Y., Hu Y. (2018). Ionic electroactive polymers used in bionic robots: A review. J. Bionic Eng..

[15] Mun S.C., Yun S.R., Nam S.W., Park S.K., Park S.T., Park B.J., Lim J.M., Kyung K.U. (2018). Electro-active polymer based soft tactile interface for wearable devices. IEEE Trans. Haptics..

[16] Qiu Y., Lu Z., Pei Q. (2018). Refreshable tactile display based on a bistable electroactive polymer and a stretchable serpentine joule heating electrode. ACS Appl. Mater..

[17] Gupta U., Qin L., Wang Y., Godaba H., Zhu J. (2019). Soft robots based on dielectric elastomer actuators: A review. Smart Mater. Struct..

[18] Hajiesmaili E., Clarke D.R. (2021). Dielectric elastomer actuators. J. Appl. Phys..

[19] Qiu Y., Zhang E., Plamthottam R., Pei Q. (2019). Dielectric elastomer artificial muscle: Materials innovations and device explorations. Acc. Chem..

[20] Ji X., Haitami A.E., Sorba F., Rosset S., Nguyen G.T.M., Plesse C., Vidal F., Shea H.R., Cantin S. (2018). Stretchable composite monolayer electrodes for low voltage dielectric elastomer actuators. Sens. Actuators B Chem..

[21] Youn J.H., Jeong S.M., Hwang H.W., Kim H.W., Hyeon K.J., Park J.W., Kyung K.U. (2020). Dielectric elastomer actuator for soft robotics applications and challenges. Appl. Sci..

[22] Ghosh S., Inganäs O. (2000). Networks of electron-conducting polymer in matrices of ion-conducting polymers applications to fast electrodes. Electrochem. Solid-State Lett..

[23] Kaneto K. (2016). Research trends of soft actuators based on electroactive polymers and conducting polymers. J. Phys. Conf. Ser..

[24] Wallmersperger T., Kröplin B., Holdenried J., Gulch R.W. A coupled multi-field-formulation for ionic polymer gels in electric. Proceedings of the Fifth World Congress on Computational Mechanics (WCCM V).

[25] Bae J.H., Chang S.H. (2019). PVDF-based ferroelectric polymers and dielectric elastomers for sensor and actuator applications: A review. Funct. Compos. Struct..

[26] Sikulskyi S., Mekonnen D.T., Atrache A.E., Divo E., Kim D.W. (2021). Effects of ferroelectric fillers on composite dielectric elastomer actuator. Actuators.

[27] Qiao B., Wang X., Tan S., Zhu W., Zhang Z. (2019). Synergistic effects of maxwell stress and electrostriction in electromechanical properties of Poly(vinylidene fluoride)-based ferroelectric polymers. Macromolecules.

[28] Choi S.T., Kwon J.O., Bauer F. (2013). Multilayered relaxor ferroelectric polymer actuators for low-voltage operation fabricated with an adhesion-mediated film transfer technique. Sens. Actuators A Phys..

[29] Kularatne R.S., Kim H., Boothby J.M., Ware T.H. (2017). Liquid crystal elastomer actuators: Synthesis, alignment, and applications. J. Polym. Sci. B Polym. Phys..

[30] Petsch S., Rix R., Khatri B., Schuhladen S., Müller P., Zentel R., Zappe H. (2015). Smart artificial muscle actuators: Liquid crystal elastomers with integrated temperature feedback. Sens. Actuators A Phys..

[31] Rosset S., Shea H.R. (2013). Flexible and stretchable electrodes for dielectric elastomer actuators. Appl. Phys. A..

[32] Lau G.K., Goh S.C.K., Shiau L.L. (2011). Dielectric elastomer unimorph using flexible electrodes of Electrolessly Deposited (ELD) silver. Sens. Actuators A Phys..

[33] Lee Y.R., Kwon H.H., Lee D.H., Lee B.Y. (2017). Highly flexible and transparent dielectric elastomer actuators using silver nanowire and carbon nanotube hybrid electrodes. Soft Mater..

[34] Shigemune H., Sugano S., Nishitani J., Yamauchi M., Hosoya N., Hashimoto S., Maeda S. (2018). Dielectric elastomer actuators with carbon nanotube electrodes painted with a soft brush. Actuators.

[35] Sahu R.K., Saini A., Ahmad D., Patra K., Szpunar J. (2016). Estimation and validation of maxwell stress of planar dielectric elastomer actuators. J. Mech. Sci. Technol..

[36] Cameron C.G., Underhill R.S., Rawji M., Jeffrey P. (2004). Conductive filler–elastomer composites for Maxwell stress actuator applications. Smart Mater. Struct..

[37] Boldini A., Porfiri M. (2022). On Maxwell stress and its relationship with the dielectric constant in the actuation of ionic polymer metal composites. J. Mech. Phys..

[38] Zhao H., Zhang L., Yang M.H., Dang Z.M., Bai J. (2015). Temperature-dependent electro-mechanical actuation sensitivity in stiffness-tunable BaTiO3/polydimethylsiloxane dielectric elastomer nanocomposites. Appl. Phys. Lett..

[39] Kim I.J., Kim D.H., Ahn B.K., Lee H.J., Kim H.J., Kim W.H. (2021). Vulcanizate structures of NR compounds with silica and carbon black binary filler systems at different curing temperatures. Elastom. Compos..

[40] Han S.W., Kim D.H., Kim S.R., Kim J.M., Mun D.Y., Morita K., Kim W.H. (2021). Effect of silane and sulfur variation on the vulcanizate structure of silica-filled styrene-butadiene rubber compounds. Elastom. Compos..

[41] Lee M.J., Park S.H., Jhee K.H., Kye H.S., Bang D.S. (2021). Electrical properties of CNT and carbon fiber filled hybrid composites based on PA66. Elastom. Compos..

[42] Duan L., Wang G.L., Zhang Y.Y., Zhang Y.N., Wei Y.Y., Wang Z.F., Zhang M. (2018). High dielectric and actuated properties of silicone dielectric elastomers filled with magnesium-doped calcium copper titanate particles. Polym. Compos..

[43] Yang D., Zhang L., Ning N., Li D., Wang Z., Nishi T., Ito K., Tian M. (2013). Large increase in actuated strain of HNBR dielectric elastomer by controlling molecular interaction and dielectric filler network. RSC Adv..

[44] Chen T., Pan L., Lin M., Wang B., Liu L., Li Y., Qiu J., Zhu K. (2015). Dielectric, mechanical and electro-stimulus response properties studies of polyurethane dielectric elastomer modified by carbon nanotube-graphene nanosheet hybrid fillers. Polym Test..

[45] Chen T., Qiu J., Zhu K., Li J., Wang J., Li S., Wang X. (2015). Achieving high performance electric field induced strain: A rational design of hyperbranched aromatic polyamide functionalized graphene−polyurethane dielectric elastomer composites. J. Phys. Chem. B..

[46] Karolina G., Xiangdong X., Stanislaw G., Roland K. (2017). Electrical, mechanical, and thermal properties of LDPE graphene nanoplatelets composites produced by means of melt extrusion process. Polymer.

[47] Amutha S.A.J., Lndhumathi K., Arun Prakash V.R. (2020). Role of cobalt nanowire and graphene nanoplatelet on microwave shielding behavior of natural rubber composite in high frequency bands. Polymer Compos..

[48] Yaguang G., Liwu L., Yanju L., Jinsong L. (2021). Review of dielectric elastomer actuators and their applications in soft robots. Adv. Intell. Syst. Comput..

