# Supplementary material for: Effect of the Particle Size and Layer Thickness of GNP Fillers on the Dielectric Properties and Actuated Strain of GNP–PDMS Composites
Source: Polymers (Basel). 2022 Sep 13;14(18):3824. doi: 10.3390/polym14183824 (PMC9502295; doi:10.3390/polym14183824)
Supplement: Supplementary file 1 [file polymers-14-03824-s001.zip › polymers-1859471-supplementary.pdf]

Supplementary

Effect of the Particle Size and Layer Thickness of GNP Fillers on the Dielectric Properties and Actuated Strain of GNP–PDMS Composites

Jin-Sung Seo [1], Do-Hyeon Kim [1], Heon-Seob Jung [2], Ho-Dong Kim [2], Jaewon Choi [3], Minjae Kim [4], Sung-Hyeon Baeck [1] and Sang-Eun Shim [1, *]

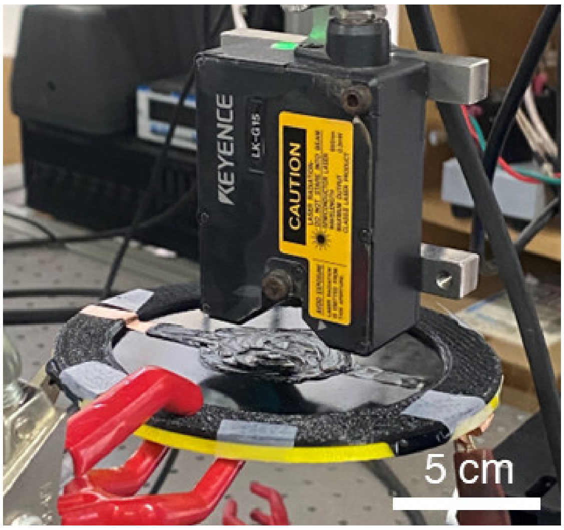

Figure S1. DEA circular test of PDMS/GNP sample using a laser sensor (laser doppler vibrometer).

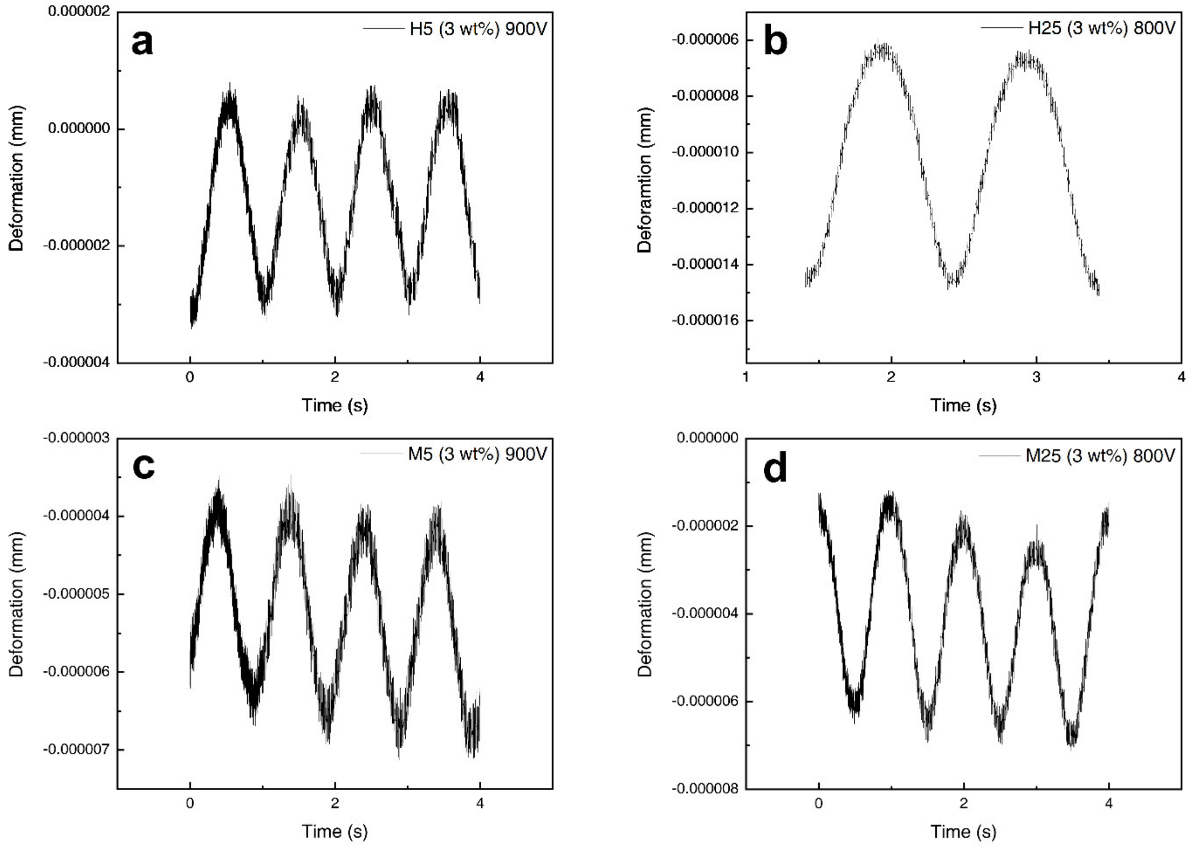

Figure S2. Deformation at maximum voltage of PDMS with 3 wt% addition of 4 grade GNPs measured with a laser doppler vibrometer.
